# Modeling the Adhesion Bonding Strength in Injection Overmolding of Polypropylene Parts

**DOI:** 10.3390/polym12092063

**Published:** 2020-09-10

**Authors:** Ruggero Giusti, Giovanni Lucchetta

**Affiliations:** Department of Industrial Engineering, University of Padova, via Venezia 1, 35131 Padova, Italy; ruggero.giusti@unipd.it

**Keywords:** overmolding, adhesion bonding, polypropylene, injection molding, healing, self-diffusion, reptation, composites

## Abstract

In this work, the bonding strength of overmolded polypropylene is investigated and modeled. A T-joint specimen was designed to replicate the bonding between a base and an overmolded stem made of the same polymer: a previously molded plaque was used for the base, and the stem was directly overmolded. The effect of melt temperature, holding pressure, and localized heating was investigated following the design of experiments approach. Both the melt and base temperature positively affect the welding strength. On the contrary, the holding pressure negatively contributed, as the crystallization temperature significantly increases with pressure. Then, the bonding strength of the specimens was predicted using a non-isothermal healing model. Moreover, the quadratic distance of diffusion (based on the self-diffusion model) was calculated and correlated with the bonding strength prediction. The non-isothermal healing model well predicts the bonding strength when the reptation time is calculated within the first 0.09 s of the interface temperature evolution. The prediction error ranges from 1% to 35% for the specimens overmolded at high and low melt and base temperatures, respectively.

## 1. Introduction

In recent years, the integration of various secondary operations in the injection molding process was proposed to satisfy the needs of the industry, primarily when the business is based on high volume production. The use of injection molding is even more interesting for the advantages in terms of high productivity and automatization, allowing the coupling of several technologies [1]. One of the main goals is to join two or more parts during the cycle, and for this purpose, the insert-molding or the overmolding process in its variants, from 2 K up to 4 K, is well known [2,3].

The joining between parts can be attained in various ways: by mechanical interlocking, where the molten material fills in undercuts on the insert; using adhesives, as in the case of the in-mold-bonding; exploiting an activation process, such as plasma or flame; or using direct welding between plastic parts [4,5,6,7,8]. The overall advantage of these processes is to reduce secondary operations, such as post-welding, riveting, and gluing. For this reason, the maximization of the strength of the direct joining is of fundamental importance. In the automotive field, the insert molding evolved into the overmolding of metal sheets for the manufacturing of polymer–metal composite parts (PMH) [9]. The base concept is the same as the insert molding, but the technology is aimed at the manufacturing of semi-structural and structural parts by using formed metal sheets.

In recent years, the regional regulation on CO_2_ reduction and end-of-life treatment, combined with the need to increase the safety standard, pushed the industry to design lightweight solutions based on metal replacement. In several applications, the metal sheet was replaced by overmolded organosheets [10,11]. In this process, a thermoplastic laminate reinforced with woven fiber is directly formed inside the mold cavity and then overmolded with reinforcing ribs to obtain a thermoplastic hybrid composite (TPHC) [12].

This variant takes advantage of the chemical affinity of the two matrices, which are of the same family, and the direct adhesion induced by the high process temperatures. In hybrid parts such as in other kinds of assembly, one of the weakest points is the joining. For example, in the overmolding of hybrid composites, the weaker area can move from the interface between matrices to inside the laminate at the matrix-woven interface. In this case, as well as in the overmolding of plastic parts, the bonding between matrices can theoretically reach the strength of the matrix, and it is fundamental to understand how the process parameters can maximize it [13].

In this work, a T-joint specimen was manufactured by using a homopolymer polypropylene: a previously molded plaque was used for the base, and the stem was directly overmolded. The effect of melt temperature and holding pressure were evaluated following the design of experiments approach, and a full factorial plane was run. In addition, the effect of the localized heating of the base before the injection phase was investigated.

The bonding phenomenon is well described by the reptation theory, which was formulated by De Gennes [14] on Edwards’ hypothesis [15]. In this study, the healing model for non-isothermal processes that was proposed by Bastien and Gillespie was used, which is based on the work of Doi and Edward [16,17]. In this model, the distance of diffusion across the interface is directly related to the bonding strength. The study was deepened by calculating the quadratic distance of diffusion, as it was proposed in recent works [18,19,20,21], and employing the self-diffusion coefficient proposed by Graessley, which is also based on the study by Edward and Doi [22]. Both the models were applied, and a relation between the two different approaches was proposed. In addition, each parameter that describes the modeling was discussed.

## 2. Theoretical Background

The bonding process between two thermoplastic materials is characterized by two main phenomena that define the degree of bonding. The first contribution is given by the intimate contact that indicates the physical contact between the two surfaces. In the weld lines that are typical of injected molded parts, where bonding occurs at temperatures close to the melting point at high pressure, it can be assumed that the intimate contact is achieved almost instantaneously when the molten flow fronts merge.

The second contribution is given by the phenomenon of self-diffusion of the macromolecules. In thermoplastic polymers, it can occur only when the macromolecules have enough energy for the motion. In particular, the self-diffusion occurs when the polymers are in contact above their glass transition temperature or the crystallization temperature for amorphous or semi-crystalline polymers, respectively.

**Figure 1:**
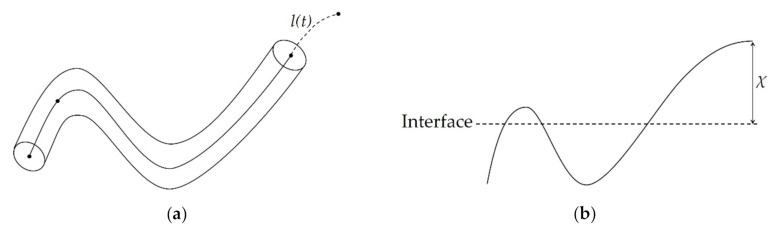
(**a**) Minor chain length *l(t)* according to the reptation theory; (**b**) Interpenetration distance of a chain across the interface. Adapted from [17].

In the interdiffusion of polymer molecules across a bonding interface, the reptation theory is used to predict the distance that is covered by a macromolecule after the time *t* (Figure 1a) [14,15]. The distance *l* is called minor chain length and is defined by Equation (1).

(1) $$\frac{l}{L}={\left(\frac{t}{t_{rep}}\right)}^{1/2},$$

where *L* is the length of the tube.

The creation of a bond can be attributed to the minor chains crossing the interface and creating entanglement, which is usually referred to as healing or autohesion. The same theory can be used to predict the distance of the average interdiffusion. This is assumed to be equal to the average distance between the end of the macromolecule and the interface. Equation (2) allows the prediction of the average distance of interdiffusion *χ*, which is represented in Figure 1b, at the time *t*.

(2) $$\frac{\chi }{\chi_{\infty }}={\left(\frac{t}{t_{rep}}\right)}^{1/4},$$

where *χ_∞_* is the radius of gyration *R_g_* of the macromolecule.

As time increases, the interface disappears, and the properties of the bulk material are reached for a time longer than the reptation time.

The healing theory, as presented by Kim and Wool [23], can relate microscopic parameters, such as *l* and *χ*, to macroscopic mechanical properties, such as strength and toughness. The model makes assumptions on the failure mechanism that can be due to chain pullout (stress-based) or to chain breakage (energy-based).

The healing model states that the fracture stress is proportional to the interpenetration distance, and, according to Equation (3), it is proportional to the fourth root of time. Based on similar considerations, the fracture energy, *G_C_*, is proportional to the square root of the minor chain length, and, according to Equation (4), it is proportional to the square root of time. The proportionalities are presented in the following equations:

(3) σσ∞=χχ∞=(lL)12=(ttrep)14,

(4) GG∞=lL=(ttrep)12,

where *σ_∞_* and *G_∞_* are the fracture stress and energy of the fully healed interface. These relations are valid for isothermal healing at a temperature *T* and for process times *t* less than or equal to *t_rep_*. To predict the fracture stress and energy, it is necessary to determine their maximum values under controlled conditions experimentally. Moreover, the reptation time, which is related to the thermal conditions, must be carefully evaluated.

The healing model used in this work for the prediction of the bonding strength is the non-isothermal healing model proposed by Bastien and Gillespie [17]. In the model development, the temporal domain of a non-isothermal healing process was divided into n time intervals, and the process in each *n*-th interval was considered to be isothermal at the average temperatures T^*^ between *t_i_* and *t_i+_*_1_.

(5) $$\Delta t=\left(t_{i+1}-t_{i}\right)=\frac{t_{healing}}{n},$$

where *t_healing_* indicates the time-domain of the model. The incremental bond strength provided within each ∆*t* was derived from the isothermal reptation theory, and the model intended to determine the bond strength at any instant t as the summation of the increments. The final expression of the non-isothermal healing model is the following:

(6) σσ∞=χχ∞=∑t=0tΔt[ti+114−ti14trep(T*)14].

In the work of Yang and Pitchumani [24], the model proposed by Bastien and Gillespie and the subsequent improvement proposed by Sonmez and Hand [25] were critically discussed. It was highlighted how the model was unsuitable for non-isothermal processes especially of high molecular weight polymers. Bastien and Gillespie’s model predicted a longer time to get the maximum degree of healing in the heating cycle, and the opposite happens in the cooling cycle, with a prevision of shorter bonding time.

The macromolecular interdiffusion between matrices can also be described as a function of the self-diffusion coefficient by calculating the quadratic distance of diffusion. Graessley proposed a self-diffusion coefficient that is based on the viscoelastic parameters of the polymer that participates in the healing [22]. The self-diffusion coefficient is described by the following equation:

(7) $$D=\;\frac{G_{N}^{0}}{135}\;{\left(\frac{\rho RT}{G_{N}^{0}}\right)}^{2}\;\left(\frac{\langle r^{2}\rangle }{M_{w}}\right)\;\frac{M_{c\;}\left(T\right)}{M_{w}^{2}\;\eta_{0,M_{c}\left(T\right)}},$$

where *G_N_*^0^ is the plateau modulus, *ρ* is the density of the polymer, *R* is the universal gas constant, *T* is the absolute temperature, <*r*^2^> is the mean square end-to-end distance of the macromolecules, *M_w_* is the molecular weight, *M_c_(T)* is the critical molecular weight at the temperature *T*, and *η_0,Mc(T)_* is the zero-shear viscosity at the critical molecular weight and temperature *T*.

The self-diffusion coefficient *D* can be used for the calculation of the quadratic distance of diffusion of the macromolecules that participate in the healing of the interface. The quadratic distance of diffusion is calculated as [18,20,21,22]:

(8) $$\langle l^{2}\rangle {}_{Z}=2\int_{0}^{t_{diff}^{Z}}D\left(t\right)dt.$$

The time of diffusion at the interface is calculated as follows:

(9) $$T_{Z}\left(t_{diff}^{Z}\right)=T_{c},$$

where *T_Z_* is the temperature of the median plane, *t_diff_^Z^* is the diffusion time at the median plane, and *T_C_* is the crystallization temperature of the polymer.

By comparing the quadratic distance of diffusion <*l*^2^> with the mean square end-to-end distance <*r*^2^>, it is possible to establish if the interdiffusion of the macromolecules has occurred. The complete healing occurs when the quadratic distance of diffusion exceeds the mean square end-to-end distance [18].

## 3. Healing Modeling

This work takes as reference the approach proposed by Lafranche for evaluating the effect of the interdiffusion at the polymer–polymer interface in polyamide–matrix hybrid specimens [19,20,21]. The self-diffusion coefficient was calculated considering the interface temperature evolution across the stem section, which was determined with a one-dimensional thermal simulation. The model is defined by a thermo-dependent factor and has a domain of application that is inferiorly limited by the crystallization temperature.

The integration of the quadratic distance of diffusion was discretized in the summation of each isothermal contribution:

(10) $$\langle l^{2}\rangle =2\sum_{t=i}^{n}D\left(T^{*}\right)(t_{i+1}-t_{i}).$$

A final comparison between the non-isothermal healing model (Equation (6)) and the self-diffusion model (Equation (7)) was carried out using Equation (11), which correlates the interpenetration distance to the mean square end-to-end distance as well as to the calculated quadratic distance of diffusion:

(11) $$R_{g}^{2}=\frac{1}{6}\langle r^{2}\rangle ,$$

where *R_g_* corresponds to the radius of gyration, *χ_∞_* [21].

Both models are based on the average temperature T^*^ of the *n*-th interval. Both the non-isothermal model for the self-diffusion calculation and the one used for the prediction of the bonding strength were applied using the time–temperature history of the interface. The temperature evolution at the interface was calculated using a numerical simulation of the injection overmolding process, which was performed using the software tool Autodesk Moldflow Insight 2019 and setting the same initial and boundary conditions that characterize the experimental process.

Since the domain of the models is lower bounded by the crystallization temperature or by the glass transition temperature (for semi-crystalline or amorphous polymers, respectively), the simulation allows determining the time domain. The interval of application considered in this work starts at the end of the filling phase and ends when the interface temperature reaches the polypropylene crystallization temperature.

The relaxation time of an amorphous material is equivalent to the reptation time [21]. For a semi-crystalline material, the assumption remains valid when the degree of crystallinity is approximately constant [24]. In applying the healing model, the critical aspect of the proposed approach was to model the reptation time between the melting and the crystallization temperature of polypropylene. For this reason, three different models were used to extrapolate the reptation time at the average temperature of the time intervals. The first one is the WLF equation:

(12) $$\log a_{T}=-\frac{A_{1}\left(T-T^{*}\right)}{A_{2}+\left(T-T^{*}\right)},$$

where the shift factor *a_T_* = *t_rep_*/*t_rep_*(T^*^). The constants of the WLF model were determined by creating the time–temperature superposition (TTS) curve of the polypropylene at a reference temperature of 220 °C. Then, the constants *A*_1_ and *A*_2_ were fitted, setting T^*^ to the crystallization temperature, and the reptation (i.e., relaxation) time at the crystallization temperature was calculated.

The same approach was followed for the second model, i.e., the Arrhenius equation:

(13) $$\log a_{T}=\frac{E_{a}}{RT},$$

where *E_a_* is the activation energy, and *k_B_* is the Boltzmann constant.

The third model uses a power law to interpolate the experimental values of the reptation time between 190 and 260 °C.

In order to apply the self-diffusion model, the plateau modulus, *G_N_*^0^, the molecular weight, *M_w_*, the critical molecular weight at the temperature *T*, *M_c_(T)*, and the zero-shear viscosity at the critical molecular weight and temperature *T*, *η_0,Mc(T)_* were determined.

The plateau modulus *G_N_*^0^ for amorphous material was obtained from dynamic rheology measurements. Several methods are proposed in the literature for the determination of such values, as reported in the work of Liu et al. [26]. Among others, the modified MAX method for polydisperse polymers (Equation (14)), which considers a shifted Rouse model for the terminal spectrum of uniform entangled space, was selected for both its simplicity and its agreement with other methods.

(14) $$G_{N}^{0}=4.83G_{max}^{\prime \prime }.$$

The value of 4.83 for the proportionality constant in Equation (14) is in accordance with several experimental results reported by Liu et al., also including some syndiotactic polypropylenes, having the constant in the range 4.69 ÷ 5.08. In this work, the value of *G_max_^”^* was assumed equal to the crossover modulus, *G_c_*.

A frequency sweep test, conducted on a rotational rheometer, allowed determining the value of *G_c_*. The critical molecular weight *M_c_*(*T*) is correlated to the molecular weight of the entanglement *M_e_* [27], where *M_e_* is correlated to the plateau modulus *G_N_*^0^ [28]. The relations are reported in Equation (15) and Equation (16), respectively.

(15) $$M_{c}\left(T\right)=2M_{e}\left(T\right),$$

(16) $$M_{e}\left(T\right)=\frac{\rho RT}{G_{N}^{0}}.$$

The mean end-to-end distance of the macromolecule, as the name indicates, is the average distance between the ends of the macromolecule, and it defines the lower limit for having a complete self-diffusion. As a result of the experimental difficulties for determining this parameter, it is common to express it in relation to the molecular weight. For polypropylene, such a relation is the following [29]:

(17) $$\frac{\langle r^{2}\rangle }{M_{w}}=\;0.694\;\;{Å}^{2}.$$

The zero-shear viscosity is related to the molecular weight by a power law [29]:

(18) $$\eta_{0}=KM_{w}^{a},$$

where the constant *K* and the exponent *a* are temperature dependent. The exponent *a* is generally lower than one when the molecular weight is lower than *M_c_*, and it is in the range 3.4 ÷ 4.2 when the molecular weight is higher than *M_c_*. To identify the parameters of Equation (18), the molecular weight and zero-shear viscosity values of two similar polypropylenes (having different *M_w_* values) were selected from the literature [30]. The values of *K* and *a* were calculated by fitting the zero-shear viscosity vs. molecular weight plot for temperatures of 190, 220, and 250 °C.

## 4. Materials and Methods

**Figure 2:**
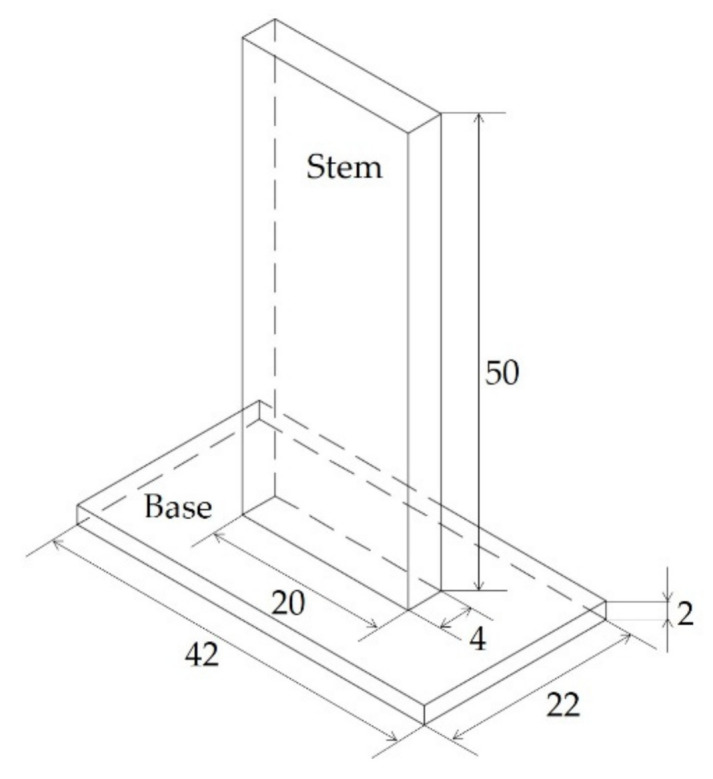
Design of the T-joint specimen. All dimensions are in millimeters.

The T-joint specimen is made of a 2 mm thick base with an area of 22 mm × 42 mm and an overmolded stem with an area of 4 mm × 20 mm and a height of 50 mm. The interface has a nominal area of 4 mm × 20 mm. The geometry is shown in Figure 2.

Both the base and the stem are made of a homopolymer polypropylene (Lukoil, Buplen 6331, Moscow, Russia). The material was characterized using both a rotational (TA Instruments, Ares, New Castle, DE, USA) and a capillary rheometer (Ceast, RHEO2000, Pianezza, Italy) for the Newtonian and pseudoplastic regions, respectively. The rotational rheometer was used in dynamic oscillatory mode at an imposed strain. Measurements were performed in frequency sweep mode (0.1 to 100 s^−1^) for temperatures between 190 and 250 °C. The capillary rheometer was used with two different dies (L/D of 0.5 and 10), and the imposed shear rate ranged from 50 to 5000 s^−1^. The data were corrected by applying Bagley’s and Rabinowitsch’s corrections. The experimental data were fitted to the Cross-WLF model.

The melting temperature, the crystallization temperature, and the glass transition temperature were determined by using a differential scanning calorimeter (TA Instruments, Q200, New Castle, DE, USA) by performing complete heating and cooling cycle with a ramp of 10 °C/min.

The characterization of the reptation time was carried out using a rotational rheometer (TA Instruments, Ares, New Castle, DE, USA), performing a frequency sweep test in the range 1–100 rad/s at temperatures between 190 and 260 °C with a step of 10 °C. The dynamic shear response was analyzed to determine the crossover frequency and calculate the relaxation time that was assumed equal to the reptation time.

A two-cavity mold was designed to realize a T-joint at the end of the filling phase. The mold is provided with a transversal beam with two cavity slots that contain the rectangular bases. The slots are perpendicular to the parting plane to allow the manual handling and the in-cavity positioning of the base after a preliminary IR heating. Further details about the experimental setup are described in [31].

All the samples were overmolded using a 1000 kN electrical injection molding machine (Engel, E-Motion 440/100, Schwertberg, Austria). The base preheating was carried out using a 200 mm × 200 mm IR lamp (Krelus, Oberentfelden, Switzerland). The base temperature was checked using a probe (Hasco Z2512, Guntramsdorf, Austria) to evaluate its cooling due to handling, which took a total of 30 s before the start of mold filling: 20 s for the handling and 10 s for the mold closure.

The tensile tests of the T-specimens were performed on a universal tensile testing machine (MTS Systems Corporation, 322, Eden Prairie, MN, USA) with a load cell of 5 kN. The base was slightly clamped between the plates of the support to avoid any bending. It was secured using a steel plate with a central rectangular hole having sides 1 mm longer than the sides of the interface area [31]. The lateral surfaces of the stem were free to slide without interference with the steel plate. This device allowed the stem clamping and prevented any kind of bulk loading of the sample during the closing of the clamps. Each test was displacement controlled with a rate of 2 mm/min and a frequency acquisition of 20 Hz.

The process parameters’ effects on the ultimate tensile strength (UTS) of the bonding were evaluated following a full factorial design with two parameters at two levels, as reported in Table 1. All the other process parameters were kept constant (Table 2). The base preheating and handling cycle was designed in order to have a surface temperature of 130 °C at the beginning of the filling phase. All the runs were repeated nine times.

The experimental plan reported in Table 1 was also run without preheating. In this case, the temperature of the base at the beginning of the filling phase was 32 °C. This value considers that the bases were initially at environmental temperature and then were heated by the mold (80 °C) during the closing time.

Numerical simulation of the injection overmolding process was conducted to calculate the temperature evolution of two overlapping interface nodes that belong to the base and the stem, respectively. The intermediate results were calculated at a constant interval to have a regular high-resolution temperature evolution.

## 5. Results

### 5.1. Bonding Strength Characterization

Table 3 reports the analysis of variance (ANOVA) conducted on the experimental plan. It indicates that both the melt temperature and the holding pressure significantly affect the bonding strength.

**Figure 3:**
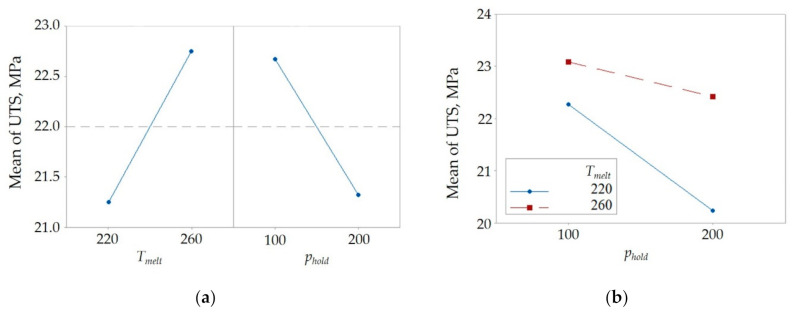
(**a**) Main effects and (**b**) interaction plots for the melt temperature and holding pressure.

The positive effect of the melt temperature (Figure 3) was expected, as a higher interface temperature significantly promotes the macromolecular interdiffusion. The negative effect of the packing pressure was unexpected, since it is known that pressure contributes to increasing the degree of contact [31]. However, as the crystallization temperature significantly increases with pressure [32], the timeframe for molecular interdiffusion reduces. The positive contribution of pressure in increasing the degree of contact saturates for a certain level of pressure. Beyond this level, the negative effect of holding pressure in increasing the crystallization temperature prevails.

**Figure 4:**
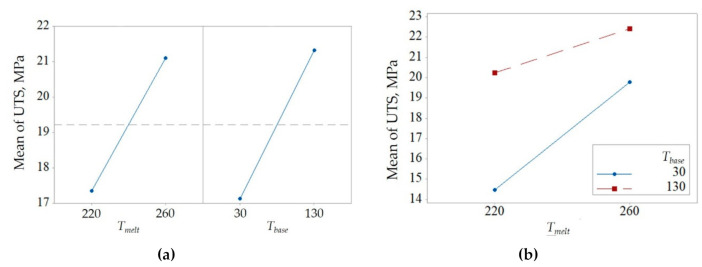
(**a**) Main effects and (**b**) interaction plots for the melt temperature and base temperature.

Table 4 reports an ANOVA considering the combined variations of melt temperature and base temperature. They both significantly and positively affect the bonding resistance. The positive contribution is also confirmed by the interaction between the two factors (Figure 4).

These results can be explained considering the macromolecular interdiffusion that is promoted by the higher temperature at the interface:

(19) $$T_{i}=\frac{T_{melt}+T_{base}}{2}.$$

When both *T_melt_* and *T_base_* are at the low level, the temperature at the interface is 125 °C and increases up to 195 °C when they are at the high level.

### 5.2. Non-Isothermal Healing Model Results

The results from the rheological and thermal characterization of the considered polypropylene are reported in Table 5 and Table 6, respectively.

**Figure 5:**
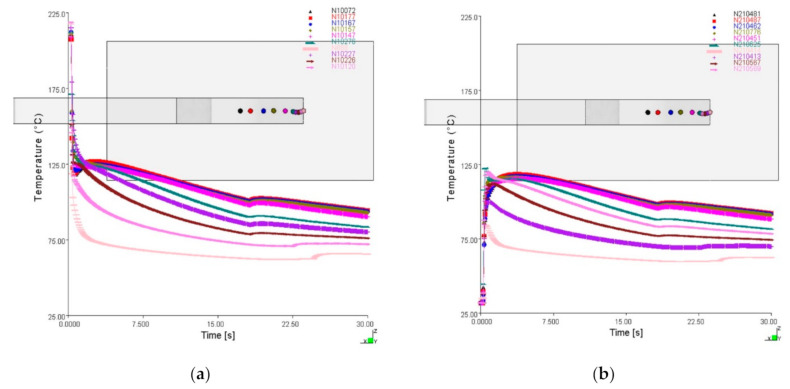
Interface temperature evolution of (**a**) the overmolded stem and (**b**) the base, as numerically calculated with both *T_melt_* and *T_base_* initially at the low level.

The average value between the interface temperature of the overmolded stem and the base (Figure 5) was calculated from the numerical simulation using Equation (19). Then, the average temperature evolution was discretized in sub-intervals of equal duration, which were characterized by constant average temperature values.

**Figure 6:**
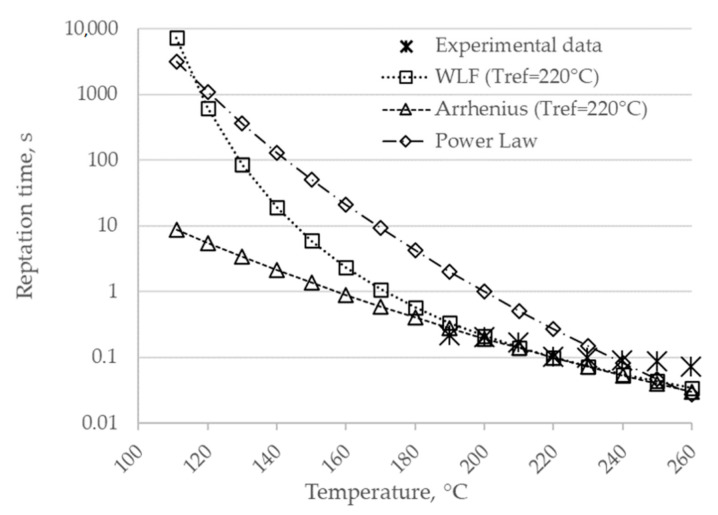
Comparison of the three models used for the extrapolation of *t_rep_* between the melting and the crystallization temperature.

Figure 6 shows a comparison between the experimental data and the three methods that were proposed to extrapolate the reptation time.

Even though both the WLF and the Arrhenius model show a good agreement in interpolating the experimental results in the range 190 ÷ 260 °C, only the WLF model shows a sudden increase of the reptation time with decreasing temperatures, which agrees with the trend proposed by Bastien and Gillespie [17]. On the other hand, the power-law model is not able to interpolate the experimental results.

**Figure 7:**
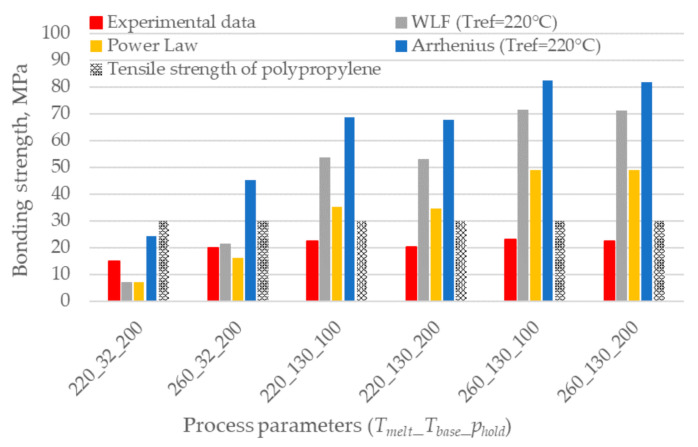
Comparison between the experimental bonding strength and the predictions based on the interface temperature evolution. The combination of process parameters is expressed using the code *T_melt_*_*T_base_*_*p_hold_*.

Then, these models were used to calculate the reptation time at the interface temperature and predict the bonding strength according to the healing model (Equation (6)). Figure 7 shows the comparison between experimental results obtained with different process parameters, analytical predictions, and the UTS of the used polypropylene. None of the models can predict the strength of the weaker specimens. This is probably due to the low accuracy in determining the reptation time at low temperatures as in the case where the base is provided at 32 °C and the melt temperature is 220 °C with a consequent interface temperature of 126 °C. On the contrary, the models overpredict the bonding strength when the temperature at the interface is higher for longer.

The models underestimate or overestimate the bonding strength for low and high base temperatures, respectively, except for the Arrhenius model, which always overestimates the results. This overprediction is related to the unsuitability of the model to extrapolate the reptation time at temperatures lower than the reference one.

As the phenomena of intimate contact and diffusion simultaneously occur when the interface temperature is high (i.e., low reptation time), the model was applied considering two shorter timeframes for the temperature evolution, 0.9 s and 0.09 s, which correspond to the contribution of the first nine time intervals and of the first single time interval, respectively.

**Figure 8:**
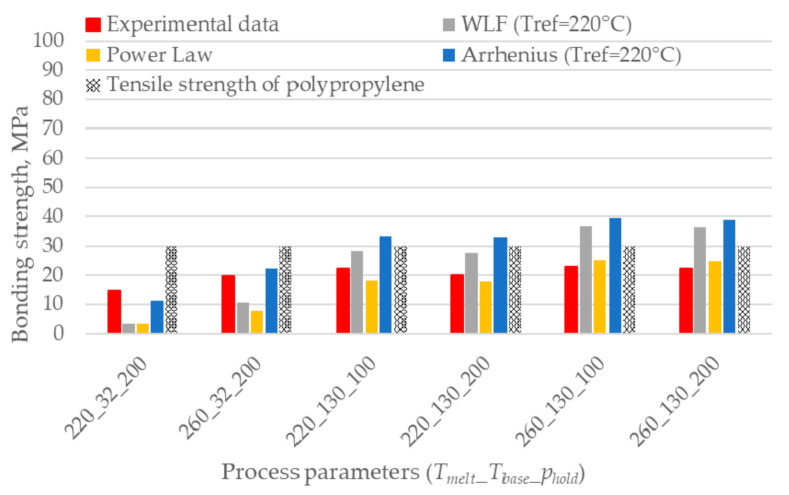
Comparison between the experimental bonding strength and the predictions based on the first 0.9 s of the interface temperature evolution. The combination of process parameters is expressed using the code *T_melt_*_*T_base_*_*p_hold_*.

Figure 8 shows that the trend is still the same, although a reduction in the prediction error is noticeable. The Arrhenius model still overestimates the result, but it now shows the same trend as the experimental results.

**Figure 9:**
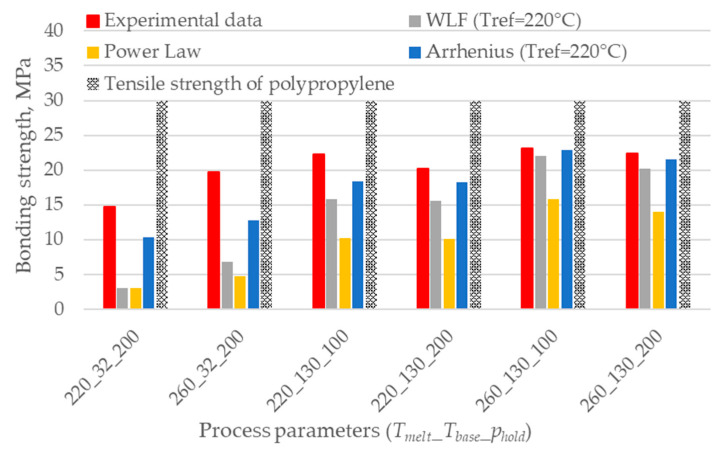
Comparison between the experimental bonding strength and the predictions based on the first 0.09 s of the interface temperature evolution. The combination of process parameters is expressed using the code *T_melt_*_*T_base_*_*p_hold_*.

Interestingly, Figure 9 shows that the healing model based on the Arrhenius model well predicts the bonding strength when the reptation time is calculated considering only the first 0.09 s of the interface temperature evolution. The reduction of the prediction error is noticeable, and its value ranges between 1% and 35% for the specimens overmolded at high and low melt and base temperatures, respectively: the latter undergo shorter cooling to crystallization.

### 5.3. Self-Diffusion Model Results

**Figure 10:**
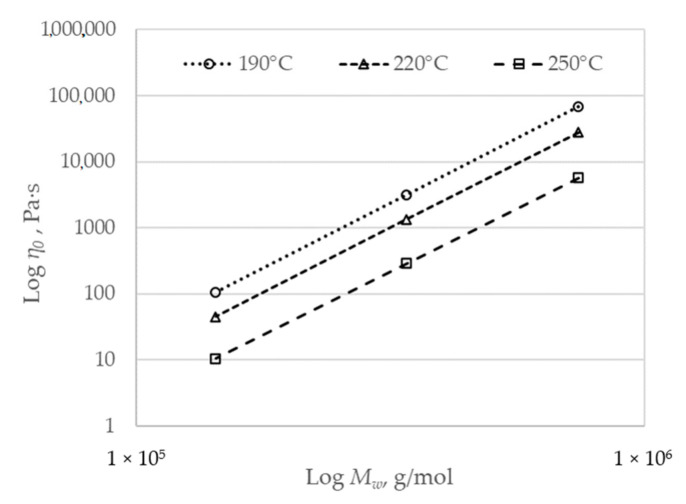
Zero-shear viscosity dependence on molecular weight at different temperatures.

Figure 10 shows the zero-shear viscosity vs. molecular weight plot for temperatures of 190, 220, and 250 °C.

The data fitting at each of the three temperatures provided three different values for *K* and *a*, respectively, which were used to fit the following power-law equations:

(20) $$K=6.776E-11T^{-3.537},$$

(21) $$a=6.776T^{-0.103}.$$

**Figure 11:**
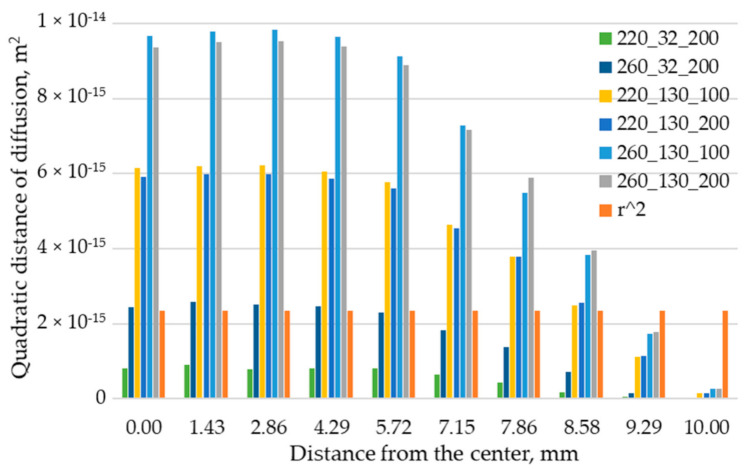
Gradient of the quadratic distance of diffusion for specimens produced with different interface temperature evolutions.

Figure 11 shows the quadratic distance of diffusion, calculated through Equation (10), plotted against the distance from the center of the stem in the interface plane.

The quadratic distance of diffusion decreases by getting closer to the mold wall (located distant from the center), where the skin acts as a barrier for the interdiffusion. Only at the lowest melt and base temperature values, the self-diffusion is incomplete, as the quadratic distance of diffusion is lower than the mean square end-to-end distance.

**Figure 12:**
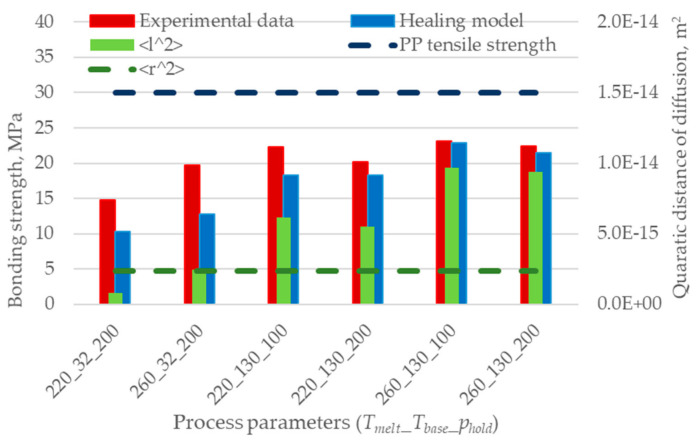
Gradient of the quadratic distance of diffusion for specimens produced with different interface temperature evolutions.

Figure 12 compares the bonding strength, as predicted by the non-isothermal healing model based on the first 0.09 s of the interface temperature evolution and the Arrhenius extrapolation (Figure 9), with the quadratic distance of diffusion at the central node of the interface. The results show a high correlation between the mobility of macromolecules (i.e., quadratic distance of diffusion) and the interface temperature evolution, which is related to the non-isothermal healing.

The results of the non-isothermal healing model and the values of the quadratic distance of diffusion are summarized in Table 7. The two different approaches are linked by Equation (11), where the interpenetration distance *χ* and the quadratic distance of diffusion <*l^2^*> are used. The *χ* values calculated using the two models are linearly correlated with a coefficient of determination R^2^ = 0.99. Moreover, the values predicted by the two models are in good accordance, especially for the specimens overmolded at high temperatures.

## 6. Conclusions

In this study, the bonding strength of overmolded polypropylene parts was experimentally investigated and analytically modeled. The effect of melt temperature, holding pressure, and localized heating was investigated following the design of experiments approach. Both the melt and the base temperature positively affect the bonding strength, as a higher interface temperature significantly promotes the macromolecular interdiffusion. On the other hand, a higher holding pressure decreases the bonding strength by reducing the timeframe for molecular interdiffusion due to the increase of the crystallization temperature with pressure.

Two different approaches to the bonding strength prediction were evaluated and compared: the non-isothermal healing model and the self-diffusion model. Both models are based on the reptation theory; therefore, they are significantly influenced by the interface temperature evolution. The non-isothermal healing model well predicts the bonding strength when the reptation time is calculated within the first 0.09 s of the interface temperature evolution. The prediction error ranges from 1% to 35% for the specimens overmolded at high and low melt and base temperatures, respectively. However, the predictions of the non-isothermal healing model are highly dependent on its formulation. This happens especially for highly non-isothermal processes because the first term of the summation gives the largest contribution. Moreover, the modeling of the reptation time is crucial. When the first time interval is considered, the formulation that better predicts the bonding strength is the one that uses the Arrhenius equation to extrapolate the reptation time between the melting and the crystallization temperature.

The quadratic distance of diffusion, calculated using the self-diffusion model and the temperature evolution of ten nodes located on the interface, showed a good correspondence with the bonding strength. The quadratic distance of diffusion decreases by getting closer to the mold wall, where the skin acts as a barrier for the interdiffusion. This result suggests that for better modeling of the bonding strength, the quadratic distance of diffusion should be averaged on the interface area.

The interpenetration distance values calculated using the non-isothermal healing model and the quadratic distance of diffusion are linearly correlated with a coefficient of determination R^2^ = 0.99. Moreover, the values predicted by the two models are in good accordance, especially for the specimens overmolded at high temperatures.

## Figures and Tables

**Table 1:** Investigated factors for the overmolding of the T-joint specimens.

Factors	Low	High
Melt temperature, °C	220	260
Holding pressure, bar	100	200

**Table 2:** Fixed injection molding parameters for the overmolding of the T-joint specimens.

Injection Molding Parameter	Value
Injection flow, cm^3^/s	63
Backpressure, bar	30
Holding time, s	35
Mold temperature, °C	80
Cooling time, s	65
Contact time before closure, s	5

**Table 3:** ANOVA table for the effect of the melt temperature and the holding pressure on the bonding strength.

Factor	Sum of Square	Mean Square	F-Value	*p*-Value
*T_melt_*	20.372	20.372	6.49	0.016
*p_hold_*	16.443	16.443	5.24	0.029
T_melt_*p_hold_	4.277	4.277	1.36	0.252
Error	100.46	3.140		

**Table 4:** ANOVA table for the effect of the melt temperature and the base temperature on the bonding strength.

Factor	Sum of Square	Mean Square	F-Value	*p*-Value
*T_melt_*	126.68	126.68	36.68	0.000
*T_base_*	159.53	159.53	46.19	0.000
*T_melt_* * *T_base_*	21.84	21.84	1.36	0.017
Error	100.52	3.141		

**Table 5:** Cross-WLF parameters of the considered polypropylene.

Parameter	Value
*n*	0.251
*τ*, Pa	3273.59
*D*_1_, Pa · s	5.15E+07
*D*_2_, K	384.45
*A*_1_	16.41
*A*_2_, K	51.6

**Table 6:** Thermal and mechanical properties of the considered polypropylene.

Property	Value
Crystallization temperature, °C	111.3
Transition temperature, °C	−18.6
Melting temperature, °C	167.5
Molecular weight, g/mol	3.40 × 10^5^
Tensile strength, MPa	30

**Table 7:** Comparison between the predictions of the non-isothermal healing model and self-diffusion model.

		Non-Isothermal Healing Model		Self-Diffusion Model	
Specimen	*σ_exp_*, MPa	*σ_pre_*, MPa	*χ*, m^2^	*<l*^2^*>*, m^2^	*χ*, m^2^
220_32_200	14.46	10.35	6.8 × 10^−9^	8.0 × 10^−16^	1.20 × 10^−8^
260_32_200	19.77	12.74	8.4 × 10^−9^	2.5 × 10^−15^	2.00 × 10^−8^
220_130_100	22.27	18.35	1.2 × 10^−8^	6.2 × 10^−15^	3.20 × 10^−8^
220_130_200	20.22	18.25	1.2 × 10^−8^	5.5 × 10^−15^	3.00 × 10^−8^
260_130_100	23.08	22.91	1.5 × 10^−8^	9.7 × 10^−15^	4.00 × 10^−8^
260_130_200	22.42	21.50	1.4 × 10^−8^	9.4 × 10^−15^	3.90 × 10^−8^
